# Burden of Treatment‐Induced Vasomotor Symptoms in Men With Prostate Cancer on Androgen‐Deprivation Therapy: A Systematic Literature Review

**DOI:** 10.1155/proc/7791468

**Published:** 2026-09-10

**Authors:** Mayank Ajmera, Kai-Jye Lou, Sydney Ng, Michele Helbing

**Affiliations:** ^1^ Astellas Pharma Global Development, Northbrook, Illinois, USA; ^2^ Analysis Group, Boston, Massachusetts, USA, analysisgroup.com

**Keywords:** activities of daily living, patient-reported outcomes, quality of life

## Abstract

**Background:**

Androgen‐deprivation therapy (ADT) for prostate cancer (PCa) is associated with vasomotor symptoms (VMS; hot flashes and night sweats). However, the impact of VMS has not been well characterized. This systematic literature review analyzed patient‐reported outcome (PRO) instruments used to assess the quality of life (QoL) impact of ADT‐induced VMS as well as the associated humanistic burden and epidemiology.

**Methods:**

MEDLINE In‐Progress, Embase, Cochrane Database of Systematic Reviews (2011–2024), and major oncology and health economics and outcomes research conferences (2023–2024) were searched.

**Results:**

In total, 52 studies were included. Of 15 PRO instruments identified, the most common were the Hot Flash‐Related Daily Interference Scale, the Expanded Prostate Index Composite‐26, and hot flash daily diaries. No PRO instrument was specifically validated to assess the effect of ADT‐induced VMS on QoL in PCa. VMS severity (including subjective burden) was significantly associated with QoL, whereas VMS frequency was not. However, no consensus exists on VMS severity definitions. VMS negatively affected sexual functioning, interpersonal relationships, and/or work productivity, and the associated anxiety, fatigue, insomnia, and poor sleep quality were found to impair QoL. VMS additionally appeared to mediate ADT’s impact on sleep disturbance. ADT‐induced VMS rates ranged from 1.4% to 68.9%, with higher frequencies reported within 3–6 months of ADT initiation and when adverse events were actively monitored or participants asked to report/record VMS events (30.7%–68.9%). Lower frequencies were seen when VMS were spontaneously captured or in retrospective analyses without active VMS monitoring/reporting (1.4%–15.8%) and with enzalutamide, apalutamide, and abiraterone as add‐on therapy to ADT (13.9%–15.8%).

**Conclusions:**

VMS severity in men with PCa is more consistently associated with QoL impact versus VMS frequency. A need for PRO instruments specific to treatment‐related VMS in PCa and a lack of consensus on definitions of severity for ADT‐induced VMS in PCa were identified.

## 1. Introduction

Androgen‐deprivation therapy (ADT) is a core component of standard care for prostate cancer (PCa), but its use is associated with vasomotor symptoms (VMS; hot flashes and night sweats) [1–3]. Hot flashes are characterized by sudden, intense sensations of heat that may be accompanied by flushing and sweating, primarily affecting the face, neck, and trunk [1, 4]. VMS in men with PCa have been associated with sleep disturbance, poor treatment tolerability, and decline in quality of life (QoL) that can lead to ADT non‐adherence and/or discontinuation [1]. Despite being a known side effect of ADT, the specific effects of ADT‐induced VMS in men with PCa are not well‐characterized. This systematic literature review (SLR) aimed to describe the available patient‐reported outcome (PRO) instruments used to assess the impact of VMS among men with PCa and improve understanding of these instruments. Additional objectives were to describe the humanistic burden of VMS among men with PCa, including the impact on QoL and activities of daily living (ADL), symptoms that are most troubling or have the most significant impact on QoL or daily functioning, and the epidemiology of VMS in PCa.

## 2. Methods

This SLR was conducted in accordance with the Preferred Reporting Items for Systematic Reviews and Meta‐analyses (PRISMA) 2020 statement [5]. Searches covered English language articles that described observational clinical studies (including real‐world evidence) and clinical trials in men with PCa receiving ADT. The PICOS (Population, Patient, or Problem; Intervention; Comparison; Outcome; Study) criteria used are described in Table 1. The following electronic databases were searched on October 24, 2024: MEDLINE and MEDLINE In‐Progress (OvidSP), Embase (OvidSP), and the Cochrane Database of Systematic Reviews (CDSR; OvidSP). The complete strategy used to search electronic article databases is shown in Table S1. A supporting hand search of major oncology and health economics and outcomes research conferences within the previous 2 years (2023–2024) was conducted to capture additional publications through November 20, 2024 (Table S2).

**TABLE 1:** PICOS criteria.

Elements	Inclusion criteria	Exclusion criteria
Interventions	• Patients with PCa receiving ADT	• Patients with PCa not receiving ADT
		• Patients with other cancers or malignancies

Comparators	• Pharmacologic ADT interventions, including:	• Other treatments not used in the management of PCa
	◦ Hormonal therapy/androgen receptor pathway inhibitors	• Nonpharmacologic ADT interventions, including:
	◦ Luteinizing hormone‐releasing hormone	◦ Chemotherapy
	◦ Gonadotropin‐releasing hormone	◦ Radiotherapy/brachytherapy
		◦ Systemic therapy
		◦ Surgery (prostatectomy)
		◦ Ultrasound/laser ablation, electroporation, photodynamic therapy

Outcomes	• Burden of treatment‐related VMS as assessed via:	• Outcomes not directly linked to treatment‐related VMS
	◦ QoL	
	◦ Activities of daily living	
	◦ Symptoms that are most troubling or reported to have the most significant impact on QoL or daily functioning	
	• Epidemiology of treatment‐related VMS:	
	◦ Incidence	
	◦ Prevalence	
	◦ Frequency	
	• PRO instruments used to assess the impact of treatment‐related VMS	

Study design	• Clinical trials	• Clinical trials
	• Cohort studies	• Case reports
	• Case–control studies	• Case series
	• Cross‐sectional studies	• Narrative reviews
	• Epidemiological studies	• Economic models
	• Longitudinal studies	• Editorials
	• Systematic literature reviews/meta‐analyses	• Letters
		• Opinions
		• Animal/preclinical studies

Sample size	Epidemiological studies: sample size ≥ 200	Epidemiological studies: sample size < 200

Language	English only	Non‐English

Publication year	Published 2011 or later	Published 2010 or earlier

Abbreviations: ADT, androgen‐deprivation therapy; PCa, prostate cancer; PICOS, Population, Patient, or Problem, Intervention, Comparison, Outcome; PRO, patient‐reported outcome; QoL, quality of life; VMS, vasomotor symptoms.

### 2.1. Screening Methodology

Two researchers (SN and DT) independently screened hits for relevance to the research questions according to PICOS criteria (Table 1). First, titles and abstracts were reviewed for inclusion or exclusion based on the PICOS criteria (Level 1 screening). Next, full texts of studies that passed Level 1 screening were screened to confirm they met the PICOS criteria and included information relevant to addressing one or more research questions (Level 2 screening). Full texts for records that could not be included or excluded with reasonable confidence during Level 1 screening also underwent Level 2 screening. Screening results were reviewed, and differences in screening decisions were adjudicated by a third researcher (KJL). Studies with sample size < 200 were excluded from the epidemiology analysis dataset to address potential confounds and limited generalizability.

### 2.2. Data Extraction

Data from studies that passed Level 2 screening were extracted independently by two researchers (SN and DT or OR) in parallel. Data extractions were reviewed and adjudicated by a third researcher (KJL). Extracted data elements included publication and study information and participant characteristics, PRO instruments assessing the impact of ADT‐induced VMS, outcomes related to burden of VMS (QoL, ADL, symptoms that were most troubling or reported to have the most significant impact on QoL or daily functioning), and incidence, prevalence, and frequency of ADT‐induced VMS.

## 3. Results

### 3.1. Search Results

**FIGURE 1:**
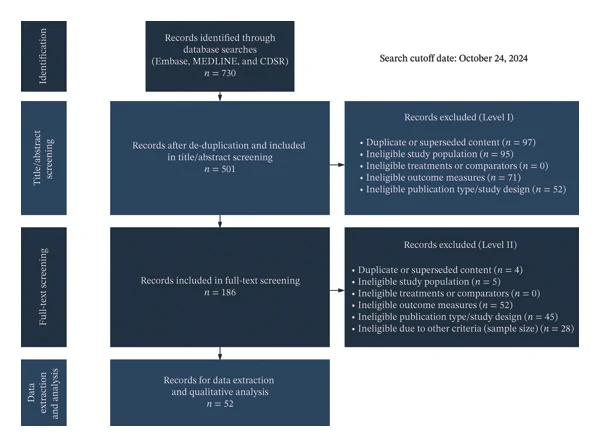
PRISMA diagram.

Article database searches returned 730 studies; no additional studies were identified through hand searches (Figure 1, Table S1), and 52 studies were selected for data extraction (38 real‐world studies and 14 clinical trials; Table S3). The number of studies identified by key topic area is summarized in Table 2.

**TABLE 2:** Article counts^a^ by topic area.

Topic area	Real‐world study	Clinical trial	Total
PRO instruments used to assess the impact of ADT‐induced VMS	15	9	24
Humanistic burden of ADT‐induced VMS	24	11	35
Epidemiology of ADT‐induced VMS	21	7	28
**Total unique studies**	**38**	**14**	**52**

*Note:* Bold text indicates the total number of studies.

Abbreviations: ADT, androgen‐deprivation therapy; PRO, patient‐reported outcome; VMS, vasomotor symptoms.

^a^Articles that covered multiple topic areas of interest were multicounted.

### 3.2. PRO Instruments Used to Assess the Impact of Treatment‐Related VMS

Fifteen PRO instruments assessing the impact of ADT‐induced VMS in men with PCa were identified across 24 studies (Table 3) [6–29]. The most common instruments were the Hot Flash‐Related Daily Interference Scale (HFRDIS; seven studies) [7–13], Expanded Prostate Index Composite‐26 (EPIC‐26; six studies) [7, 10, 14–17], and hot flash daily diaries (five studies) [10, 18–21]. HFRDIS was originally developed and validated for women with menopause and breast cancer. Hence, its validity and sensitivity for assessing the impact of ADT‐induced hot flashes in men with PCa are unclear. EPIC‐26 is a validated PRO instrument for assessing patient QoL that includes one VMS‐specific item asking men to rate “How big a problem during the last 4 weeks, if any, has hot flashes been for you?” on a scale of 0 (*no problem*) to 4 (*big problem*). However, it is not specifically designed to assess the impact of VMS on patient QoL. The designs of the Hot Flash Daily Diaries varied across the five studies employing them [10, 18–21]. A prospective, 6‐week study of 10 men with locally advanced PCa detailed the following items participants were asked to include in their daily diaries: date and time of each hot flash, severity using a scale of 0 (*not at all*) to 10 (*extreme*), and duration of each hot flash in minutes [10]. Three studies recorded general frequency and severity of hot flashes with different severity rating scales [18, 20, 21]. One study recorded sleep start/end and the number, duration, and reasons for sleep disturbances in the morning and evening [19].

**TABLE 3:** PRO instruments are used to assess ADT‐induced VMS in patients with PCa.

PRO instrument	Number of studies	Validation status	Strengths/limitations
Hot Flash‐Related Daily Interference Scale (HFRDIS)	7 (3 clinical trials [1 RCT; 2 non‐RCTs]; 4 observational studies)	Validated in women	+ Widely used instrument
			+ Evaluates impact of hot flashes across multiple domains
			− Does not assess night sweats
			− Not validated for use in men or in PCa

Expanded Prostate Index Composite‐26 (EPIC‐26)	6 (2 clinical trials [1 RCT, 1 non‐RCT]; 4 observational studies)	Validated in PCa	+ Widely used instrument in PCa
			+ Includes single VMS‐specific question on problem rating of hot flashes
			− Not specifically designed to assess the impact of hot flashes on QoL
			− Does not assess night sweats

Hot Flash Daily Diaries^a^	5 (3 clinical trials [2 RCT, 1 non‐RCT], 2 observational studies)	Validated in women^b^	+ Widely used instrument
			+ Typically includes assessments of hot flash frequency, severity, and bothersomeness
			− Not validated for use in men or in PCa
			− May not assess night sweats
			− Variability in tool design complicates comparison and generalizability across studies

Aging Males’ Symptoms	2 (1 RCT; 1 observational study)	Validated in testosterone deficiency	+ Includes an item to rate the severity of excessive sweating (including hot flushes) as well as sleep problems
			− Not specifically designed to assess the impact of hot flashes on QoL
			− No specific mention of night sweats in questionnaire
			− Not validated for use in PCa

European Organisation for Research and Treatment of Cancer Quality of Life Questionnaire‐Prostate 25‐Item (EORTC QLQ‐PR25)	2 (both RCTs)	Validated in PCa	+ Widely used instrument in PCa
			+ Includes single VMS‐specific question to rate the frequency of hot flashes on 4‐point scale
			− Not specifically designed to assess the impact of hot flashes on QoL
			− Does not assess night sweats

Hot Flush Beliefs and Behaviors Scale (HFBBS)	2 (1 RCT; 1 observational study)	Validated in women	+ Evaluates impact of hot flashes and night sweats across multiple domains
			− Not validated for use in men or in PCa

Hot Flush Rating Scale	2 (1 RCT; 1 observational study)	Validated in women	+ Evaluates frequency and problem rating of hot flashes and night sweats
			− Not validated for use in men or in PCa

Insomnia Severity Index (ISI)	2 (1 RCT; 1 observational study)	Validated in insomnia	+ Focused on assessing sleep disruption, which is a key impact of VMS in PCa
			− No VMS‐specific items were mentioned in questionnaire

Blatt–Kupperman questionnaire and Kupperman Menopausal Index	1 (RCT)	Not validated	+ Evaluates severity of a broad range of VMS
			− Tool is not validated
			− Tool is subject to methodological criticism due to weighting methodology and overlap in terminology across symptom categories

Degner’s Control Preferences Scale (time trade‐off) to rate “worst” treatment complications	1 (observational study)	Validated as measure of patients’ level of decision control	− No VMS‐specific items were mentioned in questionnaire
			− Tool is not validated in PCa

Hot Flash and Night Sweat Rating Scale	1 (non‐RCT)	Not validated	− Evaluates hot flashes and night sweats
			− Tool is not validated

22‐item Scale for Assessing Quality of Life of Prostate Cancer Patients Receiving Androgen Deprivation Therapy^c^	1 (observational study)	Validated in PCa	− Evaluation of hot flashes not included in final version

Physical Symptoms Questionnaire (adapted from Memorial Symptom Assessment Scale)	1 (RCT)	Not validated^d^	+ Assess frequency, severity, and bothersomeness across multiple symptom categories that overlap with VMS (e.g., sweats, difficulty sleeping)
			− No specific mention of hot flashes or night sweats
			− Tool is not validated

Symptoms and Impacts of Androgen Deprivation Therapy for Prostate Cancer (SIADT‐PC)	1 (observational study)	Not validated	+ Includes 2 VMS‐specific items on frequency of hot flashes and frequency of night sweats
			− Tool has not been evaluated in key PCa demographic groups (e.g., Black men, men > 65 years of age, men with metastatic disease)
			− Tool is not validated

Weekly hot flash score (each hot flash multiplied by a severity factor [1 for mild, 2 for moderate, 3 for severe, and 4 for very severe] and then added across the week)	1 (RCT)	Not validated	+ Evaluates frequency and severity of hot flashes
			− Does not assess night sweats
			− Tool is not validated

Abbreviations: ADT, androgen‐deprivation therapy; PCa, prostate cancer; PRO, patient‐reported outcome; QoL, quality of life; RCT, randomized controlled trial; VMS, vasomotor symptoms.

^a^Includes unspecified daily diaries that included patient‐reported frequency and/or severity of hot flashes.

^b^Approach is validated. Validation status of specific daily diaries is not well‐defined due to differences in the design of daily diaries.

^c^Details on development of this PRO instrument are described in Dun et al. (2017) [6]. In the final version of this instrument, the hot flash item was removed on the grounds of low factor loading. The study authors acknowledged this as a potential defect in their tool.

^d^The Memorial Symptom Assessment Scale is validated for use in patients with advanced cancer.

Ross et al. [22] conducted semistructured concept elicitation and cognitive debriefing interviews to develop the Symptoms and Impacts of Androgen Deprivation Therapy for Prostate Cancer (SIADT‐PC), which assessed hormonal therapy‐related symptoms and their effects on men with PCa. The study included 21 men with PCa treated with a gonadotropin‐releasing hormone agonist either as their current monotherapy or monotherapy for ≥ 6 months before another treatment was added (≤ 3 months prior). Participants experienced fatigue/lack of energy (91.7%), hot flashes/night sweats (66.7%), and insomnia/sleep problems (66.7%), which informed the final conceptual model items and domains. Limitations noted in the study included a need to perform psychometric validation of the instrument, low numbers of Black men and men > 65 years, and no men with metastatic disease.

Dun et al. [6] revised and performed psychometric validation of a novel 22‐item QoL scale for men with PCa receiving ADT. The study (*n* = 200) included an exploratory factor analysis, content and construct validity testing, and reliability testing. Hormone‐related symptoms domain items included hot flashes, weight gain, changed appearance, and breast tenderness. However, hot flashes resulted in factor loading < 0.40, suggesting poor correlation with domain, and were removed from the final scale, which the study authors noted as a major limitation of the instrument. Following the removal of hot flashes, the Cronbach’s alpha for internal consistency was 0.63 (*p* < 0.001), and the test–retest coefficient was 0.89 (*p* < 0.001).

PRO instruments lacking VMS‐specific items also were used to assess the impact of VMS on QoL, including the European Organisation for Research and Treatment of Cancer Quality of Life Questionnaire‐Core 30 (EORTC QLQ‐C30) [25, 30], Somatosensory Amplification Scale (SAS) [25], and Revised Life Orientation Test (LOT‐R) [25]. In addition, two studies assessed VMS with non‐PRO tools: the Sternal Skin Conductance monitoring device [30] and the Young Academic Urologists 25‐item Questionnaire (physician questionnaire) [23].

Four studies explored associations between VMS and patient global QoL [18, 24, 25, 30]. One was a prospective study in men with localized, nonmetastatic PCa receiving ADT versus non‐ADT–receiving controls, which reported a significant association between Aging Males’ Symptoms score and hot flashes [18]. In a cross‐sectional assessment of men receiving ADT to identify associations between VMS and QoL [25], patient‐reported problem ratings for hot flashes and night sweats were significantly associated with QoL, whereas frequency of hot flashes and night sweats was not. Two other studies evaluating ADT combined with an intervention to address VMS included assessments of the impact of VMS on patient QoL [24, 30]. In one, a randomized controlled trial evaluating luteinizing hormone‐releasing hormone agonists (LHRHa) versus a transdermal estradiol patch (tE2), an association between hot flashes and deterioration in global QoL (per EORTC QLQ‐C30) was observed across both arms at 6 months, with men who experienced more severe hot flashes reporting poorer QoL scores [24]. However, in a separate prospective study evaluating cognitive behavioral therapy versus standard treatment in men with PCa experiencing hot flashes following treatment, no significant differences in depressed mood and anxiety (per Hospitalized Anxiety and Depression Scale [HADS]) or global QoL (per EORTC QLQ‐C30) were reported at 6 and 32 weeks, despite observing significant differences in the reported rates of hot flashes and night sweats across study arms [30].

### 3.3. Humanistic Burden of Treatment‐Related VMS

A total of 35 studies described the humanistic burden of ADT‐induced VMS in PCa [6–16, 18–41], including nine on the impact of VMS severity on QoL [9, 13, 16, 25, 27–30, 35]. None directly evaluated the relationship between VMS and ADL.

#### 3.3.1. The Impact of VMS Severity and Frequency

Results from five studies generally showed subjective patient‐reported VMS severity was more likely associated with changes in QoL versus frequency of VMS [9, 16, 25, 30, 35]. Notably, a survey of 68 men with PCa undergoing ADT found an inverse correlation between patient‐reported problem rating for hot flashes and night sweats and QoL as measured by EORTC QLQ‐C30 (*r* = −0.34, *p* < 0.005) whereas no correlation was observed with the frequency of hot flashes and night sweats [25]. Frequency of hot flashes and night sweats were not significantly associated with patient‐reported problem rating, regardless of whether events were captured via subjective self‐reports or an objective sternal skin conductance monitoring device.

The definition of VMS severity varied, with some studies using objective or subjective captures of symptom frequency, duration, and intensity, and others using more subjective measures based on patient‐reported interference or problem rating. Instruments used to assess severity of ADT‐induced VMS included Hot Flash Daily Diaries, EPIC‐26 Question 13a, Hot Flush and Night Sweats Problem Rating scale, self‐reported HFRDIS, and clinician assessments [13, 15, 16, 18, 25, 29, 30, 35, 42]. Three studies categorized VMS severity on a mild/moderate/severe scale according to observations and patient responses; two studies most frequently categorized VMS severity as “mild” [25, 30], and one study most frequently categorized VMS severity as “moderate” [16]. Three other studies reported VMS severity on a mild/moderate/severe scale with nonstandard measures (Table 4) [18, 27, 42].

**TABLE 4:** Definitions of VMS severity across identified studies using nonstandard instruments.

Study	Definitions
Cheung et al. (2017) [18]	• Mild: 3 min in duration, light physical symptoms, no emotional symptoms, and no action needed
	• Moderate: Up to 5 min in duration, moderate physical symptoms, mild anxiety, some irritability and loss of concentration, need to use a fan, loosen clothing, and remove bedding
	• Severe: Up to 10 min in duration, physical symptoms described as feeling hotter or very hot, heavy perspiration, dizziness, nausea, shortness of breath, weakness, and extreme discomfort; moderate anxiety and irritability; need to loosen clothing, change clothing, and change bedding
	• Very severe: Up to 30 min in duration, very significant physical and emotional symptoms, and the need to change clothing, bedding, towel off, take a shower, and take rest

Getzenberg et al. (2020) [27]	• Mild: Sensation of heat without sweating
	• Moderate: Sensation of heat with sweating; able to continue activities
	• Severe: Sensation of heat with sweating; need to stop activity

Challapalli et al. (2018) [42]	• 1 (absent): No toxicity
	• 2 (mild): Less than 1 min in duration; self‐reported symptoms but not disrupting daily activities
	• 3 (moderate): Between 1 and 5 min in duration; bothersome toxicity affecting QoL but not disabling or severe enough for patients to seek intervention
	• 4 (severe): Greater than 5 min in duration; toxicity disrupting daily activities requiring medical intervention/discontinuation of ADT

Abbreviations: ADT, androgen‐deprivation therapy; VMS, vasomotor symptoms.

#### 3.3.2. The Impact of VMS on QoL

Nine studies described symptoms of VMS that had the most significant impact on QoL [9, 11, 13, 16, 20, 33, 35–37]. A prospective study in men scheduled to receive radiotherapy and ADT reported significant correlations between hot flashes (measured by HFRDIS) and anxiety, fatigue, insomnia, and sleep quality (all *p* < 0.001), but not with depression [11]. In a study of men with PCa receiving ADT and experiencing hot flashes and night sweats, anxiety and depression scores per the HADS were associated with the Hot Flush Rating Scale problem rating score [25].

Three qualitative interview studies in men with PCa generally reported a negative impact of VMS on sexual functioning, interpersonal relationships, and/or work productivity [29, 31, 33]. One study (*n* = 19) found that men defined the inability to fulfill personal expectations in terms of physical activity, work, sexual activity, and relationships as threats to their masculine ideals, for example, when hot flashes interfere with intimacy via altered sleeping arrangements with their partners, sexual activity, routine functioning, and strenuous activities [29]. In the second study (*n* = 21), behavioral responses to hot flashes (e.g., removal of clothing, toweling, fanning oneself) could affect relationships and social activities (e.g., avoiding some social situations) and were often accompanied by a sense of embarrassment [33]. Additionally, study participants often reported experiencing sleep disruption due to night sweats, which were associated with emotional responses including annoyance, irritation, and embarrassment. The third study reported that nine of 10 men interviewed described hot flashes to be challenging and embarrassing [31].

#### 3.3.3. The Impact of VMS on Sleep Disturbance and Fatigue

Seven studies reported sleep disturbance as an outcome associated with VMS and/or showed VMS may mediate the impact of ADT on sleep disturbance [8–10, 12, 24, 38, 39]. Three of these studies reported statistically significant associations between hot flashes/night sweats and sleep disturbance and that hot flashes significantly mediated the relationship between ADT exposure and sleep disturbance [8, 38, 39]. One study conducted secondary analyses of longitudinal QoL data in men with PCa receiving ≥ 6 months of ADT who wore actigraphs to monitor sleep and also self‐reported sleep disturbance and interference from hot flashes on the Insomnia Severity Index (ISI) and HFRDIS, respectively [8]. The study found hot flash interference (HFI) significantly mediated the association between receipt of ADT and subjective sleep disturbance (*p* < 0.05) but not wake after sleep onset, after controlling for group differences in medical comorbidities, education, and race. A prospective study in patients with nonmetastatic PCa reported that concurrent effects of ADT on insomnia symptoms were significantly mediated by night sweats (48.6% of the total effect), dyspnea (18.0% total effect), urinary symptoms (16.6% total effect), and pain (13.8% total effect) [39]. The third study in men with nonmetastatic PCa identified hot flash frequency (*p* < 0.01) and night sweats frequency (*p* < 0.01) as mediators in the observed relationship between ADT treatment and ISI scores [38].

Two studies reported a significant association between hot flashes and patient‐reported fatigue [12, 24]. A study of men with nonmetastatic PCa found impairment due to hot flashes, as measured by HFRDIS, was associated with fatigue (*p* = 0.002) but not insomnia after 4 months of ADT [12]. A separate randomized controlled trial comparing LHRHa and tE2 in men with locally advanced or metastatic PCa receiving ADT reported an association between severity of hot flashes and fatigue at 6 months across both study arms [24].

#### 3.3.4. Patient Attitudes About VMS

Four qualitative interview studies summarized patient attitudes toward hot flashes and night sweats [27, 29, 31, 33]. Two described men changing their lifestyles or adapting specific activities in response to hot flashes [27, 29], including a survey of 212 men with advanced PCa receiving ADT, which reported 23% felt embarrassed about having hot flashes, 16% changed their lifestyles because of hot flashes, and 16% considered stopping PCa treatment because of hot flashes [27].

Four studies described potential associations between humanistic burden/QoL and patient characteristics [11, 13, 14, 26]. One cross‐sectional survey of men with advanced or metastatic PCa suggested higher levels of physical activity (> 3 times/week) were associated with increased complaints of hot flashes versus less frequent exercise or no exercise [26]. A prospective survey of men with PCa reported living alone was associated with a “moderate‐to‐severe” reduction in QoL attributable to hot flashes, which was based on the EPIC‐26 (Question 13a) and feeling depressed (odds ratio = 3.2; *p* = 0.013) [14]. A prospective survey of men with newly diagnosed PCa reported those with cancer‐related cognitive impairment after 12 months had significantly greater HFI versus those without subjective cancer‐related cognitive impairment (*p* = 0.05; Cohen’s *d*_*c*_ = 0.76) [11]. Lastly, a study in men with PCa receiving ADT, matched cancer‐free controls, and men with PCa not receiving ADT reported the impact of ADT on HFI was greater with younger age and lower body mass index (BMI) at baseline [13]. Notably, ADT did not have a statistically significant impact on HFI at 12 months in men aged ≥ 82 years or those with a BMI ≥ 36.

### 3.4. Epidemiology of Treatment‐Related VMS

In total, 28 studies described the epidemiology of ADT‐induced VMS among men receiving ADT for PCa [10, 13, 16, 17, 23–26, 29, 34, 35, 38, 42–57].

#### 3.4.1. Frequency of VMS

**FIGURE 2:**
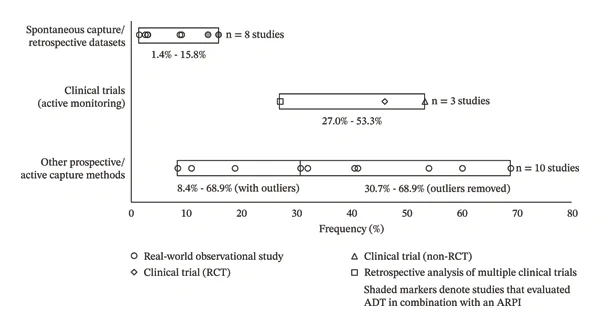
Reported frequencies of ADT‐induced VMS in patients with PCa by study type and reporting method. Includes multiple data points from studies that report the frequency of VMS among men with PCa treated with ADT across different regions, varying time points, or population subgroups. The three left‐most data points in the “other prospective/active capture methods” row appear to be outliers due to stratification to early‐stage PCa and relatively low rates of ADT use among men in the Republic of Ireland. ADT, androgen‐deprivation therapy; ARPI, androgen receptor pathway inhibitor; PCa, prostate cancer; RCT, randomized controlled trial; VMS, vasomotor symptoms.

Frequency of VMS across 21 studies ranged from 1.4% (9/652) to 68.9% (166/241) (Figure 2) [42, 54]. Higher frequencies (8.4%–68.9%) were typically reported in studies (*n* = 9) actively monitoring men for adverse events (AEs) (e.g., clinical trials) and studies prospectively recording VMS (e.g., using a daily diary, VMS‐specific survey questions) [16, 17, 24, 42, 43, 46, 47, 50, 53]. Notably, one study reporting data from multiple outlier patient groups found low hot flash frequencies in the Republic of Ireland (8.4%) and Northern Ireland (10.9%) in those with early‐stage PCa (Stage I/II), which were driven by low rates of ADT use among individuals with early‐stage PCa and, more generally, among men in the Republic of Ireland [46]. Low hot flash frequency (18.8%) also occurred among men in the Republic of Ireland with advanced PCa (Stage III/IV), who similarly had relatively low rates of ADT use versus those in Northern Ireland (where 41.1% had hot flashes). Removal of the early‐stage PCa and Republic of Ireland outlier groups (three data points) resulted in a range of 30.7%–68.9% for VMS frequency. The two studies with the highest frequency of hot flashes used survey questions around whether participants had ever experienced hot flashes (as opposed to currently experiencing hot flashes) [42, 47]. The range for VMS frequency with exclusion of both studies and the above outlier studies was 30.7%–54.0%.

Lower frequencies of VMS (1.4%–15.8%) were generally reported in studies (*n* = 7) capturing VMS spontaneously and retrospective studies analyzing datasets where active monitoring/reporting of VMS would not be expected (e.g., medical chart reviews) [44, 48, 51, 54–57]. A clinical trial reporting a 15.3% VMS frequency was in patients with PCa in a noncastrate state and, thus, was excluded from the study sets just described [49].

#### 3.4.2. Frequency of VMS According to PCa Treatment

VMS frequencies at the lower end of the range (13.9%–15.8%) were reported in men with advanced PCa in two studies of regimens that included androgen receptor pathway inhibitors (ARPIs), such as enzalutamide, apalutamide, and abiraterone [51, 56]. However, the extent to which the low frequencies were due to the treatment regimen or longer duration of exposure/acclimation to ADT is unclear, as neither study included a control arm evaluating ADT alone.

Five studies specifically described the frequency of hot flashes and/or night sweats associated with ADT in men with PCa as assessed by patient self‐report, hot flash and night sweat diary, and/or skin conductance [10, 25, 29, 34, 38]. Mean reported hot flash frequencies ranged widely from 33.32 per week to 77.7 hot flashes per day in four studies, and night sweats ranged from 18.11 to 22.2 per week in two studies [10, 25, 29, 34]. The higher end of the frequency range was in a study in men with a baseline level of VMS burden who self‐reported the frequency of hot flashes [10], whereas the lower end was in a study in men who participated in semistructured interviews and self‐reported hot flash frequency and problem ratings using a questionnaire [29]. The fifth study, a clinical trial in men with nonmetastatic PCa who assessed frequency and severity of their VMS on scales of 1 (*rarely or slight*) to 4 (*almost constantly or very severe*), reported a significant increase in hot flash frequency from baseline to 4 months and 6 months (*p* < 0.001) and in night sweat frequency from baseline to 4 months and all subsequent months (*p* < 0.05) with ADT [38].

**FIGURE 3:**
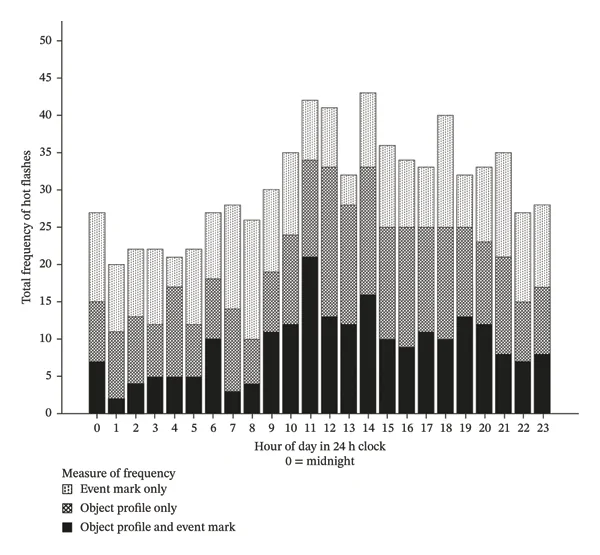
Frequency of hot flashes across 24 h. Circadian rhythm of hot flashes and activity levels among prostate cancer patients on androgen deprivation therapy. Hanisch LJ, Gehrman PR [34]. Informa UK Ltd., reprinted by permission of the publisher Informa UK Limited trading as Taylor & Francis Ltd., http://www.tandfonline.com. Note: Patients were instructed to record when they experienced a hot flash (event mark). Patients also wore devices to monitor skin conductance levels, which recorded hot flash events based on instances of a distinctive skin conductance profile that has been validated as an objective indicator of hot flash occurrence in patients with prostate cancer (objective profile). Co‐occurrences of patient‐reported and objective indicators of hot flashes were determined by an event mark immediately prior to or during an instance detected by the monitoring device (event mark and objective profile).

One study assessed the relationship between circadian rhythms in VMS in patients with PCa using both sternal skin conductance and patient‐reported data to measure hot flash episodes and found that hot flashes and activity levels tended to cluster and peak in the early afternoon (Figure 3) [34]. In addition, hot flash episodes appeared unevenly distributed over a 24‐h period and showed a circadian rhythm (*F*(2,571) = 1772.20; *p* < 0.0001), regardless of whether using patient reports (*F*(2,43) = 12.48; *p* < 0.0001) or changes in sternal skin conductance (*F*(2,43) = 43.22; *p* < 0.0001). A cosinor analysis was used to fit both a 24‐h and 12‐h rhythm to the data, with the F‐test used to assess model fit to the variance [34, 58]. No evidence of 12‐h ultradian rhythms for hot flashes was reported [34].

Two studies reported cumulative incidences of VMS [16, 52]. In one, a retrospective medical chart review of men who had received ADT ≥ 6 months, the total incidence of hot flashes was 45% [52]. The other study, a retrospective analysis observing new and cumulative longitudinal incidence after the start of ADT (index date) in men with localized PCa, found a 52.4% cumulative incidence of hot flashes over a 36‐month period in those reporting moderate/large problems [16]; VMS incidence was greatest in the first 3–6 months after ADT initiation.

## 4. Discussion

This SLR characterized PRO instruments commonly used to measure the impact of treatment‐induced VMS on QoL and summarized the humanistic burden and epidemiology of treatment‐induced VMS among men with PCa receiving ADT. The most common PRO instruments were HFRDIS (*n* = 7) [7–13], EPIC‐26 (*n* = 6) [7, 10, 14–17], and Hot Flash Daily Diaries (*n* = 5) [10, 18–21]. However, no studies validating the use of HFRDIS in men with PCa have been published, and EPIC‐26 includes only one VMS‐related item. Although hot flash daily diaries were also frequently used, implementation varied across studies. Other instruments specifically tailored to assess the impact of treatment‐related VMS on QoL included the SIADT‐PC and a novel 22‐item scale for assessing QoL of men with PCa receiving ADT [6, 22]. Notably, the SIADT‐PC is not yet validated, and the finalized version of the novel 22‐item scale removed hot flashes from the hormone‐related symptoms domain during validity testing owing to low factor loading [6]. Collectively, these observations suggest a need for the development and validation of a new PRO instrument to assess the impact of treatment‐related VMS in men with PCa. In addition, validation of existing PRO instruments that assess treatment‐related VMS in PCa is also warranted.

The SLR findings suggest VMS severity (including subjective burden) is more likely than VMS frequency to drive QoL impairment. This is because improvements in patient‐reported hot flash problem ratings tended to be associated with improved QoL [9, 16, 25, 30, 35]. However, some studies used problem ratings and interference scores to measure severity, whereas others incorporated duration and frequency metrics into their definition of severity. Overall, these observations suggest a lack of consensus around the definition of severity for VMS. Moreover, conducting robust analyses that compare outcomes across such studies will likely be challenging (and infeasible in many instances). Future efforts to build consensus in this area could include developing definitions through a Delphi panel study. Potential proposed definitions could be similar to those used for menopause‐associated VMS (i.e., mild = sensation of heat without sweating; moderate = sensation of heat with sweating, able to continue activity; severe = sensation of heat with sweating, causing cessation of activity) [59] or the scoring system developed in Challapalli et al. [42] (scores ranged from 1 = *no toxicity* to 4 = *severe*, disrupting daily activities requiring medical intervention‐discontinuation of ADT).

This SLR also identified a series of studies reporting that anxiety, fatigue, insomnia, and poor sleep quality associated with VMS can affect QoL [9, 11, 13, 16, 20, 33, 35–37]. Studies also showed that VMS may mediate the impact of ADT on sleep disturbance [8–10, 12, 24, 38, 39]. These observations are further corroborated by findings from qualitative interview studies in which men experiencing hot flashes described issues such as daytime fatigue, annoyance, irritability, and embarrassment [33, 37]. Consequently, inclusion of PRO instruments that assess treatment‐related sleep disturbance, insomnia, and/or fatigue in studies and their impact on VMS in patients with PCa may be needed. Notably, studies have reported untreated men with PCa may have disrupted circadian rhythms characterized by delayed peak androgen production [60, 61]. Hanisch et al. [34] suggested a 24‐h circadian rhythm effect of VMS is associated with ADT, with hot flash activity peaking in the early afternoon. This effect may represent a normalization of circadian rhythms following ADT. Studies have also reported cancer treatments administered in the morning are associated with significantly better QoL linked to AEs versus treatments administered in the afternoon or evening, which may also be linked with circadian clock genes [62, 63]. Additional research may characterize the relationship between ADT and circadian rhythms in men with PCa.

ADT‐induced VMS frequency ranged widely from 1.4% to 68.9% [42, 54]. Studies that actively monitored participants for AEs or specifically asked participants to report/record VMS events reported higher frequencies (30.7%–68.9% after removing data outliers) [16, 17, 24, 42, 43, 46, 47, 50, 53]. In contrast, studies capturing VMS on a spontaneous basis and retrospective analyses of datasets without the expectation of active VMS monitoring/reporting generally reported lower frequencies (1.4%–15.8%) [44, 48, 51, 54–57]. Studies of ARPIs such as enzalutamide, apalutamide, and abiraterone (as add‐on therapy to ADT) had VMS frequencies at the lower end of the observed range (13.9%–15.8%) [51, 56]. However, it is unclear whether these differences are directly attributable to the treatment regimen or to longer duration of exposure/acclimation to ADT among men with advanced PCa. Relatedly, a network meta‐analysis of various treatments for advanced/metastatic PCa by Wu et al. [64] reported a similar finding: regimens involving LHRHa were associated with higher VMS rates compared with other ADT. In particular, an analysis of the surface under the cumulative ranking curve (SUCRA) showed ADT was associated with lower rates of hot flashes (SUCRA = 89.3%) versus estrogen therapy (SUCRA = 75.5%), LHRHa (SUCRA = 32.8%), and ADT in combination with LHRHa (SUCRA = 52.5%). Additionally, a pairwise meta‐analysis found ADT alone tended to have lower risk of hot flashes when compared with LHRHa and ADT in combination with LHRHa.

The current SLR did not identify studies directly evaluating the impact of VMS on ADL, such as personal hygiene, dressing, toileting, ambulation, and eating. In addition, published evidence describing the impact of VMS on treatment patterns and on adherence and compliance in PCa is limited. Although this SLR identified a survey reporting that 16% of men considered stopping their PCa treatment because of hot flashes, this does not provide an objective assessment of the actual impact on treatment patterns or adherence/compliance [27]. These evidence gaps point to potential areas for future research to better characterize the effect of VMS on treatment patterns in PCa.

This SLR has a number of limitations. The observed heterogeneity in the definition of VMS severity across identified studies, including the incorporation of frequency metrics in select instances, represents a potential confounding factor across studies evaluating the extent to which severity or frequency of VMS drives QoL impact. Whether certain AEs were secondary to VMS or to treatment‐related AEs unrelated to VMS was often unclear. For example, although fatigue and dizziness may stem from VMS, these are also common AEs associated with additional treatments to manage PCa and comorbid health conditions. Studies describing such AEs in the PCa setting do not typically stratify them into VMS‐related and non‐VMS–related subgroups, which would make analyses of the impact of ADT‐induced VMS in this context challenging. Interpretability of the findings reported here is subject to the validity of the PRO instruments and other tools used to assess the impact of treatment‐related VMS in men with PCa. Published evidence validating their use in this setting is limited or entirely absent for most instruments and tools mentioned in this review. The implications of the SLR findings for clinical practice remain limited. The findings are descriptive in nature, and there was a lack of published evidence evaluating the impact of VMS on key clinical outcome measures in PCa as well as on treatment adherence and persistence. Furthermore, potential bias may have been introduced by the involvement of study sponsor authors who provided input on research questions, on terms used for literature searches, and on the criteria used for screening studies.

## 5. Conclusions

This SLR found no PRO instruments that were specifically validated for the effect of ADT‐induced VMS on QoL in men with PCa. The instruments most commonly used were HFRDIS, EPIC‐26, and hot flash daily diaries. Evidence suggests VMS severity in men with PCa is more consistently associated with QoL impact versus frequency of VMS. There is a lack of consensus around the definition of VMS severity in those with PCa.

## Funding

This study was funded by Astellas Pharma Inc.

## Ethics Statement

This article is a systematic literature review based on published studies and, as such, does not require institutional review board approval.

## Conflicts of Interest

Mayank Ajmera and Michele Helbing are employees of Astellas Pharma Inc. Kai‐Jye Lou is an employee of Analysis Group, Inc., a consulting firm that has received professional fees from the study sponsor. Sydney Ng is a former employee of Analysis Group, Inc., a consulting firm that has received professional fees from the study sponsor.

## Supporting Information

Additional supporting information can be found online in the Supporting Information section.

## Supporting information


**Supporting Information** TABLE S1 | Search filters and article database hits. TABLE S2 | Major oncology and health economics and outcomes research congresses searched by hand (2023–2024). TABLE S3 | Study characteristics of included studies.

## Data Availability

The data used in this systematic literature review were extracted from the existing studies cited in the manuscript, which are available in the public domain; however, some are behind a paywall and require a fee for access. The data extracted from each study are described in Table S3.
