# Supplementary material for: Burden of Treatment‐Induced Vasomotor Symptoms in Men With Prostate Cancer on Androgen‐Deprivation Therapy: A Systematic Literature Review
Source: Prostate Cancer. 2026 Sep 10;2026:7791468. doi: 10.1155/proc/7791468 (PMC13559472; doi:10.1155/proc/7791468)
Supplement: Supplementary file 1 [file PROC-2026-7791468-s001.pdf]

Burden of Treatment-Induced Vasomotor Symptoms in Men With Prostate Cancer on Androgen-Deprivation Therapy: A Systematic Literature Review

Mayank Ajmera,[1] Kai-Jye Lou,[2] Sydney Ng,[2] Michele Helbing[1]

[1]Astellas Pharma Global Development, Northbrook, IL, USA
[2]Analysis Group, Boston, MA, USA

Supplementary Material

TABLE S1 | Search filters and article database hits.

Search	Term	Number of Hits
1	*PC/ or *prostate tumor/ or *prostatic neoplasm/ or *prostatic neoplasms/	294 646
2	(("prostate" or "prostatic") adj2 ("neoplasm$" or "cancer" or "tumor" or "tumour")).ti,ab.	397 141
3	or/1-2	443 628
4	*Hot Flashes/ or *hot flush/	5095
5	("hot flash*" or "hot flush*" or "palpitation*" or "chill$" or "dizziness" or "faint*" or "sweat*" or "vasomotor" or "vaso-motor" or "VMS").ti,ab.	264 373
6	or/4-5	265 123
7	*Quality of Life/ or *Patient Reported Outcome Measures/	294 128
8	("burden" or "impact*" or "outcome*" or "patient reported" or "PRO" or "quality of life" or "QoL" or "HRQoL" or "HRQL" or "humanistic burden" or "ADL" or "activities of daily living" or "sleep" or "asleep" or "fatigue*" or "tired*" or "energy" or "mood" or "depression" or "depressed" or "anxiety" or "stress*" or "embarras*" or "cognitive" or "cognition" or "concentration").ti,ab.	19 586 104
9	("EPIC-50" or "EPIC-26" or "expanded PC index composite" or "FACT-P" or "FACT-G" or "FACT-BP" or "Functional Assessment of Cancer Therapy*" or "FAPSI" or "FPSI" or "FACIT*" or "European Organi?ation for Research and Treatment of Cancer" or "EORTC*" or "QLQ-C30" or "EuroQol*" or "EQ-5D" or "short form-36" or "SF-36" or "short form-12" or "SF-12" or "short form survey" or "Patient-Reported Outcome Measurement Information System" or "PROMIS" or "Sexual Health Inventory for Men" or "SHIM" or "Prostate Cancer Index" or "PCI" or "International Prostate Symptom Scale" or "IPSS" or "International Index of Erectile Function" or "IIEF*" or "aging male symptom*" or "AMS" or "erection sufficient for intercourse" or "ESI" or "HFRDIS" or "Hot Flash Related Daily Interference Scale" or "HFI" or "hot flash interference" or "Hot Flush Behavior Scale" or "HFBS" or "Hot Flush Rating Scale" or "Hunter" or "Kupperman" or "MENQOL" or "Daily Hot Flash Diary" or "Daily Diary" or "Work Productivity and Activity Impairment" or "WPAI").ti,ab.	495 768
10	("manag*" or "standard of care" or "SOC" or "patient journ*" or "natural histor*" or "treatment pattern*" or "pattern of treat*" or "patterns of treat*" or "pattern of thera*" or "patterns of thera*").ti,ab.	4 839 531
11	("economic*" or "cost*" or "health care financ*" or "health care management*" or "healthcare utili?ation" or "health care utili?ation*" or "health resource utili?ation" or "health service utili?ation" or "inpatient admission*" or "hospital charge*" or "resource use" or "expenditure*").ti,ab.	2 818 412
12	*absenteeism/ or *presenteeism/ or *sick leave/ or *efficiency/	32 701
13	("productivity" or "resource utili?ation" or "absen*" or "presen*" or "work disabil*" or "sick leave" or "sick day*").ti,ab.	17 619 911
14	*treatment adherence/ or *compliance/ or *treatment discontinuation/ or *persistence/	41 005
15	("compliance" or "adheren*" or "persisten*" or "discontinu*").ti,ab.	2 269 771
16	*prevalence/ or *incidence/ or *severity/	152 732

17	("epidemiolog*" or "inciden*" or "risk associat*" or "prevalen*" or "frequenc*").ti,ab.	8 149 861
18	or/7-17	38 529 517
19	("enzalutamide" or "abiraterone" or "apalutamide" or "darolutamide" or "NHT" or "hormonal therap*" or "hormone therap*" or "LHRH" or "GNRH" or "bicalutamide" or "nilutamide" or "flutamide" or "leuprolide" or "degarelix" or "relugolix" or "goserelin" or "triptorelin" or "histrelin" or "ADT" or "androgen deprivation" or "docetaxel" or "cabazitaxel" or "mitoxantrone" or "capecitabine" or "cyclophosphamide" or "estramustine" or "chemotherapy" or "sipuleucel*" or "radium*" or "ra223" or "ra-223" or "radio*" or "radiat*" or "brachytherapy" or "systemic" or "surgery" or "surgical" or "prostectomy" or "ablation" or "focal therap*" or "cryo*" or "ultrasound" or "HIFU" or "electroporat*" or "photodynamic" or "PDT" or "laser").ti,ab.	11 949 134
20	("address" or "bibliography" or "case report" or "narrative review" or "interview" or "lecture" or "legal case" or "legislation" or "patient education handout" or "letter" or "note" or "editorial" or "comment" or "addresses" or "books" or "chapter" or "legal cases").pt.	5 596 466
21	("pharmacokinetic*" or "pharmacodynamic*" or "in silico" or "in vitro" or "in vivo model*" or "mice" or "mouse" or "rodent*" or "murine" or "feline*" or "canine*" or "monkey*" or "macaque*" or "primate*" or "nonhuman" or "non-human" or "animal" or "xenograft*" or "preclinical" or "pre-clinical" or "case report*" or "rare case" or "rare presentation" or "novel case" or "novel presentation").ti.	3 910 410
22	("case report*" or "case study").ab.	665 870
23	("year old" adj1 ("male" or "man" or "female" or "woman" or "patient")).ti,ab.	1 192 357
24	("breast" not "prostate").ti.	823 742
25	letter/ or Case Study/ or comment/ or historical article/ or news/	5 913 135
26	or/20-25	13 531 747
27	(3 and 6 and 18 and 19) not 26	1075
28	limit 27 to English language	1022
29	remove duplicates from 28	731
**30**	**limit 29 to yr="2011 -Current"**	**501**

Databases included Embase, MEDLINE and MEDLINE In-Progress, and Cochrane Database of Systematic Reviews (OvidSP). Search date was October 24, 2024.

TABLE S2 | Major oncology and health economics and outcomes research congresses searched by hand (2023–2024).

**Congresses**
• American Society of Clinical Oncology (ASCO; ASCO-GU)<br>• American Association for Cancer Research (AACR)<br>• American Urological Association (AUA)<br>• European Association of Urology (EAU)<br>• European Society of Medical Oncology (ESMO)<br>• European Organisation for Research and Treatment of Cancer and National Cancer Institute and American Association for Cancer Research Symposium (EORTC-NCI-AACR)<br>• Society of Urologic Oncology (SUO)<br>• Academy of Managed Care Pharmacy (AMCP)<br>• The Professional Society for Health Economics and Outcomes Research (ISPOR)

TABLE S3 | Study characteristics of included studies.

Study	Study design	Country or region	Enrolled population (sample size)
Booth 2023 [43]	Real-world observational study	Australia	Men who received external beam RT with ADT for localized PCa (*N* = 234)
Bultijnck 2024 [26]	Real-world observational study	Belgium	ADT-treated patients with advanced or metastatic PCa (*N* = 312)
Bultijnck 2016 [23]	Real-world observational study	Europe	European physicians with experience prescribing ADT (surgical castration or luteinizing hormone-releasing hormone antagonists or LHRHa) for ≥ 6 months (*N* = 489)
Challapalli 2018 [42]	Real-world observational study	UK	Patients attending the uro-oncology clinic at Imperial College Healthcare NHS Trust, who were actively receiving ADT for PCa (*N* = 241)
Chen 2024 [7]	Prospective clinical trial	US	Radical prostatectomy (*N* = 504)
Cheung 2017 [18]	Real-world, prospective (case-control), observational study	Australia	Cases: newly commencing long-term ADT (within 6 weeks of commencement) (*N* = 63)
de Freitas 2020 [44]	Real-world, observational, prospective study	Brazil	Men with PCa, aged ≥ 18 years old, and treated with ADT (*N* = 932)
De 2022 [28]	Real-world, observational, prospective (case-control) study	US	Adult men with localized, node-negative, intermediate-risk prostate adenocarcinoma who had undergone dose-escalated EBRT (75- to 79-Gy total dose) within the last 2 years (*N* = 40)
Dieperink 2012 [14]	Real-world observational study	Denmark	PCa patients treated with 3D-CRT from January 1, 2006, to December 31, 2008, at Odense University Hospital, Denmark (*N* = 317)
Downing 2019 [17]	Real-world observational study	UK	Men alive 18–42 months after PCa diagnosis from national population-based cancer registries in England, Northern Ireland, and Wales (*N* = 35 823)
Dun 2017 [6]	Real-world observational study	China	PCa patients diagnosed from June 2008 to June 2015 at the Peking University People’s Hospital (*N* = 200)
Eziefula 2013 [29]	Real-world observational study	UK	Men with PCa experiencing hot flushes after receiving ADT for PCa (*N* = 19)
Fang 2023 [45]	Real-world observational study	US	Adverse event reports for patients receiving apalutamide were extracted from the US Food and Drug Administration Adverse Event Reporting System (*N* = 7959)

Gavin 2016 [46][a]	Real-world observational study	Ireland	Men with invasive PCa in the Republic of Ireland and Northern Ireland 1995–2010 (*N* = 3348)
Gavin 2015 [47][a]	Real-world observational study	Ireland	Men diagnosed with invasive PCa (January 1, 1995–March 31, 2010) and alive at March 31, 2011, identified through population-based cancer registries (*N* = 3348)
Gay 2017 [15]	Real-world observational study	US	Early-stage (stage T1 or T2) PCa recruited from 2003 to 2006 from the PROST-QA consortium, treated with neoadjuvant ADT (*N* = 100)
Geiges 2023 [48]	Real-world observational study	Germany	Men with PCa treated with degarelix by 93 office-based urologists (*N* = 421)
George 2022 [31]	Real-world observational study	UK	Men with advanced PCa on the treatment pathway for GnRH agonists from the uro-oncology clinic in a large teaching hospital in London (*N* = 10)
Getzenberg 2020 [27]	Real-world observational study	US	Men with advanced PCa on ADT (*N* = 212)
Gilbert 2017 [24]	Clinical trial (RCT)	UK	Patients with locally advanced or metastatic PCa and a treatment plan for indefinite ADT in the metastatic setting or ≥ 3 years for locally advanced disease (*N* = 727)
Gonzalez 2015 [13]	Real-world observational study	US	Adult men with PCa receiving ADT (*N* = 206)
Gonzalez 2018 [8]	Real-world observational study	US	Patients with PCa who were treated with ADT, previously recruited into a longitudinal QoL study (*N* = 285)
Gravis 2014 [49]	Clinical trial (RCT)	France	Patients with non-castrate metastatic PCa (NCMPC) (*N* = 385)
Grunfeld 2017 [32]	Real-world observational study	UK	Patients from the treatment arm of the MANCAN trial (*N* = 20)
Grunfeld 2012 [33]	Real-world observational study	UK	Patients with metastatic PCa from a clinic database at a large London teaching hospital (*N* = 21)
Guo 2022 [50]	Clinical trial (non-RCT)	China	New cases of HVD-mHSPC in the specialized disease outpatient departments of Minhang Hospital (*N* = 153)
Hanisch 2011 [34]	Real-world observational study	US	Patients with PCa with ongoing treatment with ADT, recruited from local oncology and urology clinics, PCa support groups and advertisements from June 2004 to February 2006 (*N* = 47)
Hanisch 2011 [19]	Real-world observational study	US	PCa survivors with ongoing treatment with ADT, recruited from the medical oncology clinic of the Abramson Cancer Center or PCa support groups, advertisements, and referrals from local urologists (*N* = 60)
Hunter 2016 [25]	Real-world observational study	UK	English-speaking men aged ≥ 18 years, undergoing ADT and having hot flushes and night sweats (minimum weekly frequency of 10) (*N* = 68)

Hussain 2022 [51]	Real-world observational study	US	US patients with physician-diagnosed nmCRPC treated with apalutamide or enzalutamide (*N* = 699)
Masuda 2014 [52]	Real-world observational study	Japan	Patients who were diagnosed with PCa by prostate needle biopsy and had taken ADT for at least 6 months (*N* = 121)
Morgans 2022 [9]	Clinical trial (non-RCT)	US	Adults with self-reported history of PCa and bothersome hot flashes recruited via social media from PCa support groups, e.g., An Can, Us TOO (*N* = 44)
Myers 2023 [35]	Clinical trial (non-RCT)	US	Men receiving ADT for PCa (*N* = 15)
Myint 2020 [53]	Clinical trials (other)	Various	Men with PCa treated by ADT-investigational agent therapy (*N* = 464)
Noorvash 2023 [36]	Real-world observational study	US	PCa patients initiating ADT and recruited from Cedars-Sinai and the Durham VA (*N* = 25)
Ohlmann 2012 [56]	Real-world observational study	Germany	Patients were eligible for the compassionate-use program if they progressed on or after at least one cytotoxic chemotherapy regimen (*N* = 398)
Pembroke 2024 [37]	Real-world observational study	Australia	PCa survivors and their partners (all female), recruited from a larger cross-sectional survey investigating cognitive functioning in PCa survivors between January 21, 2021, and October 15, 2023 (*N* = 11)
Rich 2017 [10]	Clinical trial (non-RCT)	US	Men with locally advanced PCa scheduled for proton beam treatment at Hampton University Proton Therapy Institute, Hampton, VA, who agreed to be screened for hot flash symptoms (*N* = 10)
Ross 2024 [22]	Real-world observational study	US	Adult males with PCa recruited via two recruitment agencies and one large US clinical practice specializing in PCa, and approached via secure email, telephone, or direct messaging through electronic medical record systems (*N* = 21)
Rozet 2024 [54]	Real-world observational study	France	Patients ≥ 60 years with histologically confirmed PCa, eligible to start GnRH agonist therapy before the study (*N* = 652)
Russell 2022 [20]	Clinical trial (RCT)	Australia	Males with PCa undergoing treatment with ADT (*N* = 78)
Sadikov 2016 [57]	Real-world observational study	Canada	Patients with PCa (*N* = 169)
Savard 2013 [38]	Clinical trial (non-RCT)	Canada	Participants recruited June 2004 to February 2006 at the Department of Radio-oncology at L'Hôtel-Dieu de Québec (Centre hospitalier universitaire de Québec) as part of a larger study on the role of ADT in the development of depression (*N* = 60)
Savard 2015 [39]	Real-world observational study	Canada	Patients with PCa recruited at the Centre Hospitalier Universitaire Québec from January 2005 to May 2007 (*N* = 263)
Shah 2024 [16]	Real-world observational study	US	Patients with histologically confirmed localized PCa treated with a combination of short-course ADT and SBRT and had ≥ 3 years of follow-up (*N* = 122)

Shekar 2013 [40]	Clinical trial (RCT)	NR	Patients with PCa on ADT for > 2 months (*N* = 31)
Stefanopoulou 2015 [30]	Clinical trial (RCT)	UK	PCa patients recruited by nurse specialists and medical staff from oncology and urology clinics in London, UK, April 2012 to October 2013 (*N* = 68)
Swami 2024 [55]	Real-world observational study	US	Adult patients with mCSPC from the claims database who received ADT-based treatments who had evidence of adverse events while on treatment (*N* = 4145)
Tulk 2023 [11]	Real-world observational study	Canada	Newly diagnosed men with PCa from the Dr. H. Bliss Murphy Cancer Centre (St. John's, Newfoundland and Labrador, Canada) who are aged > 18 years, fluent in written and oral English, and free of severe cognitive impairments based on screening with the mini-mental state examination (score ≤ 24) (*N* = 24)
Van Wijk 2019 [12]	Real-world observational study	Canada	Men diagnosed with non-metastatic PCa who had not yet begun ADT (*N* = 24)
Volk 2014 [41]	Real-world observational study	US	Patients with PCa nearing completion of a 6-month course of ADT at a large comprehensive cancer care center (*N* = 11)
Yu 2015 [21]	Clinical trial (RCT)	US	Men with advanced PCa receiving leuprolide depot (*N* = 51)

Abbreviations: 3D-CRT, three-dimensional conformal radiation therapy; ADT, androgen-deprivation therapy; EBRT, external beam radiotherapy; GnRH, gonadotropin-releasing hormone; HVD-mHSPC, high-volume disease metastatic hormone-sensitive prostate cancer; LHRHa, luteinizing hormone-releasing hormone agonists; mCSPC, metastatic castration-sensitive prostate cancer; nmCRPC, non-metastatic castration-resistant prostate cancer; NR, not reported; PCa, prostate cancer; QoL, quality of life; RCT, randomized clinical trial; RT, radiotherapy; SBRT, stereotactic body radiotherapy.

[a]Publications are in the same study population.
